# Early metabolic profiling in the periparturient period reduces the occurrence of postparturient metabolic diseases in cows

**DOI:** 10.5455/javar.2022.i596

**Published:** 2022-06-28

**Authors:** Mustak Ahammed, Mohammed Nooruzzaman, Md. Taohidul Islam, Md. Rafiqul Alam, Emdadul Haque Chowdhury

**Affiliations:** 1 Department of Pathology, Faculty of Veterinary Science, Bangladesh Agricultural University, Mymensingh, Bangladesh; 2 Department of Medicine, Faculty of Veterinary Science, Bangladesh Agricultural University, Mymensingh, Bangladesh; 3 Department of Surgery and Obstetrics, Faculty of Veterinary Science, Bangladesh Agricultural University, Mymensingh, Bangladesh

**Keywords:** Cows, metabolic profiling, milk fever, ketosis, mastitis

## Abstract

**Objective:**

To study the impact of early metabolic profiling and intervention measures in the periparturient period on the occurrence of postparturient metabolic diseases in cows.

**Materials and Methods:**

Using a cohort of dairy cows from two selected areas of Bangladesh, we routinely tested the serum calcium level and ketone bodies in the urine at periparturient periods. In addition, milk samples were tested for the presence of mastitis at different stages of lactation. Animals showing reduced serum calcium levels, high ketone bodies in the urine or the presence of clinical and subclinical mastitis received appropriate therapeutic intervention immediately after detection. After the intervention, the number of animals that got sick with diseases or conditions like milk fever, ketosis, mastitis, and dystocia, which are caused by metabolic problems, was recorded.

**Results:**

In the periparturient period, most of the animals had lower serum calcium levels (8.13 ± 1.2 mg/dl), which were significantly increased by the following intervention (10.05 ± 1.4 mg/dl). On the other hand, there was a higher number of ketosis-affected animals (33.9%, 20/59) during the periparturient period, which decreased (18.6%, 11/59) during the postparturient period. Similarly, the number of mastitis-affected cows was also decreased in postparturient cows (30.9%, 13/42) than in periparturient cows (59.5%, 25/42), following improved hygienic measures. After early intervention, the number of study animals with metabolic diseases decreased from 51.7% before intervention to 15.3% after intervention.

**Conclusions:**

Early metabolic profiling significantly reduced the occurrence of metabolic diseases in cows. Therefore, we recommend regular metabolic profiling of dairy cows and receiving early intervention measures to reduce the occurrence of metabolic diseases on the farm.

## Introduction

The global burden of infectious diseases in animals has been reduced during the last four decades owing to increased sanitation and preventive measures. Despite that, many production diseases in livestock species affect the sustainability of animal farming, leading to severe economic losses [1]. Disease outbreaks in dairy cows pose both financial burdens (value of the dead cow, decreased production, and extra labor) and compromised animal welfare (suffering before death or euthanasia) [2]. The diseases or conditions resulting from defects in metabolic processes are termed “metabolic diseases.” Common metabolic diseases in lactating cows include ketosis, milk fever, and downer cow syndrome [3,4]. Metabolic diseases are associated with significant economic losses due to a significant decrease in milk yield and impaired reproductive performance in animals [5,6].

Hypocalcemia in animals occurs when the blood calcium level drops to 10 mg/dl or less. In addition, dairy cows in early lactation are at a high risk of developing ketosis. It is estimated that 7–14% of dairy cows develop ketosis during early lactation, although this can vary between farms and can exceed 14% in some farms [7]. In particular, the first 2 weeks of lactation are the peak periods of developing ketosis in animals. The global prevalence of dystocia in dairy cattle is generally less than 5%, apart from those in the United States, where the prevalence of dystocia is higher than the rest of the world [8]. In Bangladesh, mastitis is one of the major limitations in successful dairy farming, with an overall prevalence of 19.9% and 44.8% of mastitis cases in dry and wet seasons, respectively [9]. A recent study using a larger cohort of dairy cows from the Chottogram district of Bangladesh showed a 43.9% incidence rate of clinical mastitis [10]. Metabolic diseases can produce acute, temporary, but potentially fatal conditions in animals. But metabolic diseases in animals could be less of a problem if they could be found and treated quickly and early during the time before and after parturition [11].

Dairy farming is a rising industry in Bangladesh. One of the major constraints of the dairy industry involving high-yielding dairy cattle is metabolic diseases like dystocia, milk fever, ketosis, and mastitis. A recent study in cattle detected a 2.82% prevalence of metabolic disorders in cattle [12]. Among the metabolic diseases, the prevalence of weak calf syndrome (1.43%) was recorded as higher than milk fever (0.95%) and grass tetany (0.44%) [12]. Another study on crossbred dairy cows also recorded a high (30%) prevalence of milk fever (30%) and ketosis (25%) [13]. These problems can easily be prevented by regular surveillance of metabolic diseases and taking prophylactic measures in time [4]. In line with this, a recent study showed improved blood calcium and glucose levels in milk fever and ketosis with the oral supplementation of calcium and propylene glycol, respectively [13]. But very limited studies have been carried out on the early rapid diagnosis and control of common metabolic diseases of dairy cattle in Bangladesh. The goal of this study was to use routine tests to find metabolic diseases in cows that were about to give birth and then take the right steps to prevent common metabolic diseases during the postpartum period. 

## Materials And Methods

### Ethical approval

The study does not involve procedures that could potentially lead to pain or distress in animals. The study was conducted as per the recommendation of the Animal Welfare and Experimentation Ethics Committee (AWEEC) of Bangladesh Agricultural University, Mymensingh (AWEEC/BAU/2020(43) dated 27.12.2020).

### Study areas

The study was conducted in two Upazilas, namely Fulbaria of Mymensingh and Nakla of Sherpur districts of Bangladesh.

### The survey

Using a structured questionnaire, we conducted a baseline survey on the incidence of metabolic diseases, such as dystocia, milk fever, ketosis, and mastitis, in the study areas from April 2016 to March 2017. After the intervention, metabolic diseases were tracked until the end of the study in April 2021.

### Sample collection and processing

Paired blood samples (*n* = 230) were collected from 115 cows in the periparturient (*n* = 115) and postparturient (*n* = 115) periods. 10 ml of blood was collected from the jugular vein of cows using disposable syringes and transferred to a tube immediately. The tube was kept in an oblique position in a cool and dry place for 2 h for the blood to clot. After 2 h, the serum was separated and collected in a clean tube. The serum was then clarified by centrifuging at 3000 rpm for 10 min, and the supernatant was collected. In addition, 118 paired urine samples were collected from 59 cows in both periparturient (*n* = 59) and postparturient (*n* = 59) periods. We collected 42 milk samples from cows at different stages of their lactation to detect the number of mastitis-affected animals. 

### Measurement of serum calcium level

The serum calcium level was determined using a Calcium liquid color kit (Human Gesellschaft fur Biochemica and Diagnostica GmbH, Wiebaden, Germany) on the Spectronic Genesys^TM^-5 (Spectronic Instrument, Inc., Rochester, NY).

### Detection of ketone bodies in the urine

The level of ketone bodies in the cows’ urine samples was measured using a rapid test kit (KetoTest, Sanwa Kagaku Kenkyusho Co. Ltd., Nagoya, Japan). Briefly, 250 ml of urine was collected directly in a dish during urination. Then, the rapid test strip was immersed in the urine and kept on a plain surface for 45 sec. After that, we determined the level of ketone bodies in the urine by comparing the color of the test strip with the standard color ranges marked on the body of the test strip pot.

### Monitoring of the somatic cell count for subclinical mastitis

We routinely collected milk samples from cows at different stages of lactation and monitored the subclinical mastitis by using the BAU Mastitis Test (BAUMT), developed by the Population Medicine and AMR Laboratory, Department of Medicine, Bangladesh Agricultural University. The interpretation and grading of the BAUMT were made by following the parameters mentioned in Table 1.

### Preventive measures

The biosecurity measures of the studied farms were increased by creating awareness and providing training to the farmers. Moreover, we offered sanitizer and handmade sanitizer spray machines to the farmers for easy administration of sanitizer on the floor and farm premises and on the hands of milkers and cows. Besides, we provided mastitis preventive therapy to periparturient or parturient cows that showed high milk leucocyte counts per the standard recommended procedure in Bangladesh. Cows that showed increased ketone bodies in urine during lactation were supplemented with 60% easily digestible carbohydrates such as green succulent grasses and a gradual increase in the amount of concentrate diet as required. To mitigate the calcium deficiency in the hypocalcemia cases, a calcium supplement with available commercial preparations at an average dose rate of 20 gm/100 kg body weight daily for 10–12 days was provided.

### Data analysis

The data were analyzed using GraphPad Prism version 5.0. Paired *t*-test was used to compare the serum calcium level in the animal cohort before and after the intervention. A *p*-value ≤ 0.05 was considered statistically significant.

## RESULTS AND DISCUSSION

### Improvement in serum calcium level

**Figure 1.**
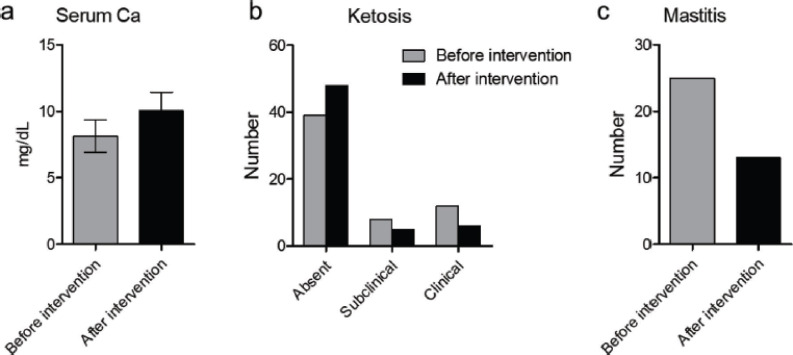
Effect of the early metabolic profiling of cows in the periparturient periods on the blood calcium level improvement and reduction in the number of ketosis and clinical mastitis cases in the postparturient periods. (a) Bar diagram showing the level of serum calcium levels; (b) the number of subclinical and clinical ketosis cases; and (c) the number of mastitis cases in cows before and after taking appropriate intervention measures as mentioned in the methodology.

The average calcium level in 115 periparturient cows was 8.13 ± 1.2 mg/dl, which was below the normal serum calcium level (9.5–10.5 mg/dl) [14]. However, following calcium supplementation during the periparturient period, the serum calcium level of these cows improved greatly, with an average of 10.05 ±1.4 mg/dl in the postparturient period (Fig. 1a). As a result, the incidence of metabolic diseases related to calcium deficiency, such as dystocia, uterine and vaginal prolapse, retained placenta, agalactia, milk fever, etc., was also reduced (Table 2).

### Reduction in the ketone body level

Urine samples (*n* = 59) from 59 cows during the periparturient period were analyzed, and subclinical and clinical ketoses were detected in 8 and 12 animals, respectively, leading to a 33.9% positive rate. The ketosis-affected cows were supplemented with 60% carbohydrate and a gradual increase in the amount of concentrate diet, which resulted in the reduction of subclinical (*n* = 5) and clinical (*n* = 6) ketoses in these animals in the postparturient period (18.6%) (Fig. 1b). 

### Reduction in the somatic cell count (subclinical mastitis)

We collected 42 milk samples from mastitis-suspected animals at different stages of lactation, and a somatic cell count was performed to detect the number of subclinical mastitis in the animals. Out of the 42 milk samples, 25 (59.5%) showed mastitis (score ≥1) with somatic cell counts of ≥400,000. Both subclinical and clinical mastitises were reduced in study animals after proper management and preventive measures, including improving hygiene. The number of mastitis cases was reduced to 13 (30.9%) (Fig. 1c). 

### The impact of early metabolic profiling on the incidence of metabolic diseases in cows

We conducted a baseline survey on the incidence of metabolic diseases, such as dystocia, milk fever, ketosis, and mastitis, in the study areas from April 2016 to March 2017. We then monitored and tested these cows regularly during periparturient periods and early lactation for serum metabolites, such as calcium and ketone bodies, as well as the presence of mastitis. After early detection of these metabolic diseases by the ways mentioned above, appropriate prophylactic measures were taken to reduce the incidence of clinical metabolic diseases (Table 2). It was noticed that the prevalence of metabolic diseases decreased from 51.7% before intervention to 15.3% during the intervention periods (May 2017–April 2021). Metabolic diseases and disorders have decreased significantly (70.4%) over the last 4 years.

**Table 1.** Interpretation and grading of the BAUMT score.

Test appearance	Results	Leukocyte count per milliliter	Grading	No. of cases
Mixture remains liquid, no precipitate	-	≤200,000	Negative	7
Slight precipitate tends to disappear with paddle movement	±	200,000–400,000	Trace	10
Distinct precipitate without a tendency toward gel formation	+	400,000–1,200,000	1	9
Distinct precipitate formation	++	1,200,000–50,000,000	2	7
Strong gel formation that tends to adhere to the paddle. Forms a distinct central peak that remains projecting above the mass	+++	>50,000,000	3	9

**Table 2.** Prevalence of metabolic diseases/disorders in cows at before and after intervention.

Diseases	Before intervention		After intervention		Remarks
	No. of cases (*N* = 145)	Percent	No. of cases (*N* = 235)	Percent	% Reduction
Dystocia	13	8.97	5	2.13	76.26
Milk fever	17	11.72	8	3.43	70.28
Ketosis	09	6.21	4	1.70	72.63
Mastitis	36	24.83	19	8.09	67.42
Overall	**75**	**51.7%**	**36**	**15.3%**	**70.4**

Sustainable dairy development in low- and middle-income countries (LMICs) necessitates coordinated efforts to address challenges in dairy production systems, such as feed, management, health, and food safety [15]. Challenges affecting sustainable dairy development in LMICs include inadequate feeding and management, poor genetics due to insufficient artificial insemination coverage, ineffective dairy processing industry, and sociocultural and demographic factors [15]. Controlling metabolic diseases is one of the important challenges in dairy farm management, and the profitability of a dairy farm relies mostly on the effective control of metabolic diseases in high-yielding cows [16]. Despite a variety of adaptation responses to nutrient and energy deficits among dairy cows, early and noninvasive detection of developing metabolic disorders in milk samples would be useful [17]. Metabolic profiling of animals by proteomics and metabolomics approaches is of great value to characterize proteins and metabolite assets from tissue and biological fluids, such as milk, blood, and urine. However, considering the limitations of small-scale dairy farmers in the LMICs, like Bangladesh, profiling essential metabolites, such as Ca and ketone bodies, would be rational. Through metabolic profiling, we could draw a forecast about the possible incidence of metabolic diseases, and we advised farmers accordingly [18]. So, we could take the necessary steps to prevent diseases, and the number of diseases went down by a lot [18].

Dystocia is a parturition disorder that has also been reduced due to better health management and proper calcium levels at the terminal stage of parturition [19]. The proper calcium level is required to maintain the strength of the uterine musculature. Prolonged hypoglycemia leads to the onset and development of clinical ketosis. Insufficient quality food intake during early lactation to meet the energy output in milk leads to a negative energy balance in animals [20]. Any stress that results in lower feed intake also results in the onset of clinical ketosis. Considering these, we advised farmers to provide 60% fresh grass and introduce concentrate feed a few weeks before parturition. Particular attention was given to the cow that experienced ketosis in her last pregnancy. Milk fever usually reduces the appetite of cows, and proper calcium supplementation reduces the incidence of ketosis, dystocia, and milk fever. The California Mastitis Test guided proper hygienic measurement and helped in controlling mastitis. 

After the early detection of subclinical conditions of metabolic diseases, we took proper intervention measures to prevent the further incidence of clinical metabolic diseases in the cow. It was noted that the prevalence of diseases was reduced greatly from 51.7% at the beginning of the study to 15.3% at the end of the study. Traditionally, the treatment of clinical mastitis includes prolonged use of antibiotics, steroids, and hyaluronidase together with oral supplementation of calcium and other minerals. Unfortunately, animals cured of clinical mastitis lose most of their production efficiency and quality of milk owing to the mammary glands’ gradual fibrosis of the glandular tissues and loss of their secretory capacity. However, early prophylactic measures can reduce subclinical mastitis, promote quicker recovery, prevent tissue damage, and improve the response to antibiotics. In turn, this improves the health status of animals and the quality and quantity of milk production.

## Conclusion

Early detection of metabolic diseases during the periparturient period helps in early intervention in affected cows. Such an early intervention significantly improves blood metabolic profiles and reduces the incidence of metabolic diseases in animals. Therefore, we recommend regular monitoring of blood profiles in dairy animals, particularly in the periparturient period, to reduce the onset of metabolic diseases in the farm. Further studies on the complete proteomic and metabolic profiling of cows at periparturient periods would be useful in better understanding and controlling metabolic diseases in dairy cows. 

## List of abbreviations

BAUMT, BAU Mastitis Test; Ca, Calcium; LMICs, low- and middle-income countries; mg, milligram; dl, deciliter
